# Integrated work processes between community health workers and vector control agents: a scoping review

**DOI:** 10.1590/0034-7167-2025-0170

**Published:** 2026-08-21

**Authors:** Karina Santos da Silva, Nakita Maria Komori, Fernanda Araújo de Paula Delfino, Kellen Campos Castro Moreira, Bethania Ferreira Goulart

**Affiliations:** I Universidade Federal do Triângulo Mineiro. Uberaba, Minas Gerais, Brazil; II Secrataria Municipal de Saúde. Uberaba, Minas Gerais, Brazil

**Keywords:** Community Health Workers, Primary Health Care, Work, Health Personnel, Review., Agentes Comunitarios de Salud, Atención Primaria de Salud, Trabajo, Personal de Salud, Revisión.

## Abstract

**Objectives:**

to map the available evidence on integrated work processes between community health workers and vector control agents in primary health care.

**Methods:**

this scoping review was conducted in November 2024 using the Cochrane Library, Embase, the Latin American and Caribbean Health Sciences Literature database, PubMed, Scopus, and Web of Science.

**Results:**

nine documents met the inclusion criteria-five articles, one conference proceedings abstract, one e-book, one monograph, and one law-underscoring the importance of professional integration to reduce fragmentation in primary health care.

**Final Considerations:**

the limited number of publications indicates a substantial evidence gap. Integration between these two professional groups may strengthen public health actions and support more efficient vector control efforts, while also contributing to responses to other public health priorities.

## RESUMEN

**Objetivos:**

mapear la evidencia sobre la integración del trabajo entre agentes comunitarios de salud y agentes de control de vectores en la atención primaria de salud.

**Métodos:**

revisión de alcance realizada en noviembre de 2024 en Cochrane Library, EMBASE, Literatura Latinoamericana y del Caribe en Ciencias de la Salud (LILACS), *National Library of Medicine* (PubMed), Scopus y *Web of Science*.

**Resultados:**

el corpus, compuesto por nueve documentos -cinco artículos, un resumen en actas de congreso, un libro electrónico, una monografía y una ley-, puso de manifiesto la importancia de la integración profesional para superar la fragmentación de la atención primaria de salud.

**Consideraciones Finales:**

la escasez de publicaciones sugiere una laguna significativa. La integración de ambas categorías profesionales puede fortalecer las acciones de salud y favorecer un enfoque más eficiente para el control de endemias y otros problemas de salud pública.

## INTRODUCTION

Primary health care (PHC), which organizes and reorients health services, serves as the population’s point of entry into the Unified Health System (Sistema Único de Saúde-SUS) and aims to address the most common, less complex health problems. The Family Health Strategy (Estratégia Saúde da Família-ESF) follows a care model grounded in determinants of health and disease and considers individuals within their family, social, economic, and cultural contexts, while also incorporating health surveillance and health promotion activities. ESF is delivered by multiprofessional PHC teams that include, at a minimum, one physician, one nurse, one nursing assistant or nursing technician, and community health workers (Agente Comunitário de Saúde-ACS), assigned to a defined catchment area^(1,2)^. ESF requires a minimum of four ACSs to ensure 100% population coverage of the catchment area, and ACSs account for more than 50% of the ESF workforce^(3)^.

In 2006, with the enactment of Law No. 11,350, the roles of ACS and vector control agents (Agente de Combate às Endemias-ACE) were regulated, and the law already envisaged joint work between the two groups^(4)^.

ACSs play a central role in health promotion in the daily lives of users and families in the areas they serve. By building rapport with users and their families, ACS staff facilitate health actions and strengthen community mobilization^(5)^.

ACEs play an equally important role in the surveillance, prevention, and control of diseases associated with environmental risk factors^(6,7)^. When their work is integrated with ACSs, ACEs can support the implementation of disease control measures during outbreaks and epidemics, including environmental management and integrated management of control activities^(8)^.

The Ministry of Health (Ministério da Saúde) recommends incorporating ACEs into ESF teams to foster collaborative work, given the need to strengthen health promotion and disease prevention by understanding and transforming the territories served^(6,7)^. This approach requires moving beyond existing fragmented, poorly integrated models and can open new possibilities for these professionals and for health managers across levels of care. Despite these guidelines, in many settings ACEs remain assigned to endemic disease control centers or to municipal epidemiological surveillance units, coordinated by professionals who are not directly part of PHC^(9,10)^.

Therefore, coordinating the work of ACSs and ACEs is essential to promote more comprehensive, problem-solving health care. Integrating their actions enables a fuller and better coordinated response to population needs, spanning health promotion, disease prevention, and endemic disease surveillance and control. Overcoming fragmentation and investing in integrated professional training are essential to strengthening health care and supporting more comprehensive care. Such integration should preserve each professional group’s specific scope of practice and facilitate the sharing of experience^(10,11)^.

## OBJECTIVES

To map the available evidence on integrated work processes between community health workers and vector control agents in primary health care.

## METHODS

### Ethical aspects

Because this review relied exclusively on publicly available data and involved no direct human participation, it was exempt from Research Ethics Committee review.

### Study design

This scoping review was conducted according to a protocol registered with the Open Science Framework (OSF; DOI: 10.17605/OSF.IO/S8ZY2) and followed Joanna Briggs Institute (JBI) guidance^(12)^ and Arksey and O’Malley’s framework^(13)^. Reporting adhered to the Preferred Reporting Items for Systematic Reviews and Meta-Analyses Extension for Scoping Reviews (PRISMA-ScR) checklist^(14)^ and comprised the following stages: defining the guiding question, identifying relevant studies, selecting studies, charting the data, and collating, summarizing, and reporting the results^(15)^.

### Identifying the guiding question

The review question was developed using the PCC framework (Population, Concept, Context): P = ACS and ACE; C = integrated work processes; and C = PHC. Accordingly, the review question was: What scientific evidence is available on integrated work processes between ACS and ACE in PHC?

### Identifying relevant studies

Data collection was conducted in November 2024 across six health databases: Cochrane Library, Embase (Elsevier), Latin American and Caribbean Health Sciences Literature (LILACS), PubMed (National Library of Medicine, National Institutes of Health), Scopus (Elsevier), and Web of Science (WoS). These sources were selected because they index a large volume of primary health research, supporting both breadth and quality of retrieval. We also searched the grey literature on official institutional websites, including those of the World Health Organization (WHO), the Pan American Health Organization (PAHO), and the Brazilian Ministry of Health. We consulted catalogs of theses and dissertations as well as national and international guidelines and resolutions. Searches across all sources were standardized through the CAPES Journals Portal (Portal de Periódicos da CAPES), accessed via the Federated Academic Community (CAFe), with access provided by a federal higher education institution.

### Study selection

Study selection followed a rigorous process that included screening titles, abstracts, and index terms to identify records that matched the predefined criteria. To maximize both sensitivity and specificity, we developed source-specific search strategies. These strategies combined Health Sciences Descriptors (DeCS) and Medical Subject Headings (MeSH) using the terms “*Agentes Comunitários de Saúde*”, “*Atenção Primária à Saúde*”, “*Trabalho*”, “*Doenças Endêmicas*”, “Community Health Workers”, “Primary Health Care”, “Work”, and “Endemic Diseases”, along with their respective synonyms, applying the Boolean operators “AND” and “OR” (Chart 1)

**Chart 1:** Search strategies used across databases for the scoping review, Uberaba, Minas Gerais, Brazil, 2025

Information source	Advanced search strategies	(n) Records retrieved
LILACS	mh:”*Agentes Comunitários de Saúde*” OR (ACS) OR (Community Health Workers) OR (*Agentes Comunitarios de Salud*) OR mh:M01.526.485.067.080$ OR mh:N02.360.067.080$ AND mh:”*Atenção Primária à Saúde*” (Primary Health Care) OR (*Atención Primaria de Salud*) OR mh: N04.590.233.727$ OR mh: D011320$ AND mh:”*Trabalho*” OR (Work) OR (*Trabajo*) OR mh:I03.946$ OR mh: D014937$ OR mh:” *Doenças Endêmicas*”	1202
PUBMED	((“Community Health Worker[Mesh] OR (Community Health Worker) OR (Health Worker, Community) OR (Health Workers, Community) OR (Worker, Community Health) OR (Workers, Community Health) OR (Community Health Aides) OR (Aide, Community Health) OR (Aides, Community Health) OR (Community Health Aide) OR (Health Aide, Community) OR (Health Aides, Community) OR (Barefoot Doctors) OR (Barefoot Doctor) OR (Doctor, Barefoot) OR (Doctors, Barefoot) OR (Family Planning Personnel) OR (Personnel, Family Planning) OR (Planning Personnel, Family) OR (Family Planning Personnel Characteristics) OR (Village Health Workers) OR (Health Workers, Village) OR (Health Worker, Village) OR (Workers, Village Health) OR (Worker, Village Health) OR (Village Health Worker)) AND (“Primary Health Care”[Mesh] OR (Care, Primary Health) OR (Health Care, Primary) OR (Primary Care) OR (Care, Primary) OR (Primary Healthcare) OR (Healthcare, Primary))) AND (“Work”[Mesh] OR (Endemic Diseases) OR (Disease, Endemic) OR (Diseases, Endemic) OR (Endemic Disease))	222
WEB OF SCIENCE/ SCOPUS	“Community Health Workers” OR “Community Health Worker” OR “Health Worker, Community” OR “Health Workers, Community” OR “Worker, Community Health” OR “Workers, Community Health” OR “Community Health Aides” OR “Aide, Community Health” OR “Aides, Community Health” OR “Community Health Aide” OR “Health Aide, Community” OR “Health Aides, Community” OR “Barefoot Doctors” OR “Barefoot Doctor” OR “Doctor, Barefoot” OR “Doctors, Barefoot” OR “Family Planning Personnel” OR “Personnel, Family Planning” OR “Planning Personnel, Family” OR “Family Planning Personnel Characteristics” OR “Village Health Workers” OR “Health Workers, Village” OR “Health Worker, Village” OR “Workers, Village Health” OR “Worker, Village Health” OR “Village Health Worker” AND “Primary Health Care” OR “Care, Primary Health” OR “Health Care, Primary” OR “Primary Care” OR “Care, Primary” OR “Primary Healthcare” OR “Healthcare, Primary” AND “Work” OR “Endemic Diseases” OR “Disease, Endemic” OR “Diseases, Endemic” OR “Endemic Disease”	508/ 524
EMBASE	‘Primary health care’/exp AND ‘ Community Health Workers’/exp AND ‘Work’/exp OR ‘ Endemic Diseases’/exp (all feeds)	0
COCHRANE LIBRARY	Community Health Workers AND Primary Health Care AND Work OR Endemic	108
Other search methods	“*Agentes Comunitários de Saúde*”, “*Atenção Primária à Saúde*”, “*Trabalho*”, “*Doenças Endêmicas*”, “Community Health Workers”, “Primary Health Care”, “Work” e “Endemic Diseases”	79
**Search date: 11/16/2024.**		

### Data charting

A total of 2,564 records were retrieved from the six databases searched, and 79 documents were identified from other sources. The PRISMA flow diagram was used to document the study inclusion process^(14)^. After retrieval, records were imported into Rayyan for screening and duplicate removal.

To minimize selection bias, two reviewers (primary and secondary) independently screened titles and abstracts against the predefined criteria and then compared their screening decisions to identify discrepancies. Disagreements were resolved by consensus; when needed, a third reviewer arbitrated.

Data extraction followed the JBI data charting tool and captured article identification, year and study setting, methodological characteristics, assessment of methodological rigor, and notes relevant to this review’s thematic focus.

### Collating, summarizing, and reporting the results

To support critical appraisal of the sources, we classified the studies’ levels of evidence and grades of recommendation according to the JBI methodology^(16)^. Levels of evidence ranged from 1 to 5, and grades of recommendation from A to C; thus, studies were classified from 1A (highest methodological quality) to 5C (lowest). We also checked the impact factor of the journals in which the studies were published using Journal Citation Reports (JCR).

Extracted information was organized in a table to facilitate synthesis. This approach supported a broad descriptive analysis, including a detailed summary of each primary study included in the review. Two experienced researchers worked collaboratively and met regularly for in-depth discussions to ensure consistency and accuracy of the results.

## RESULTS

**Figure 1:**
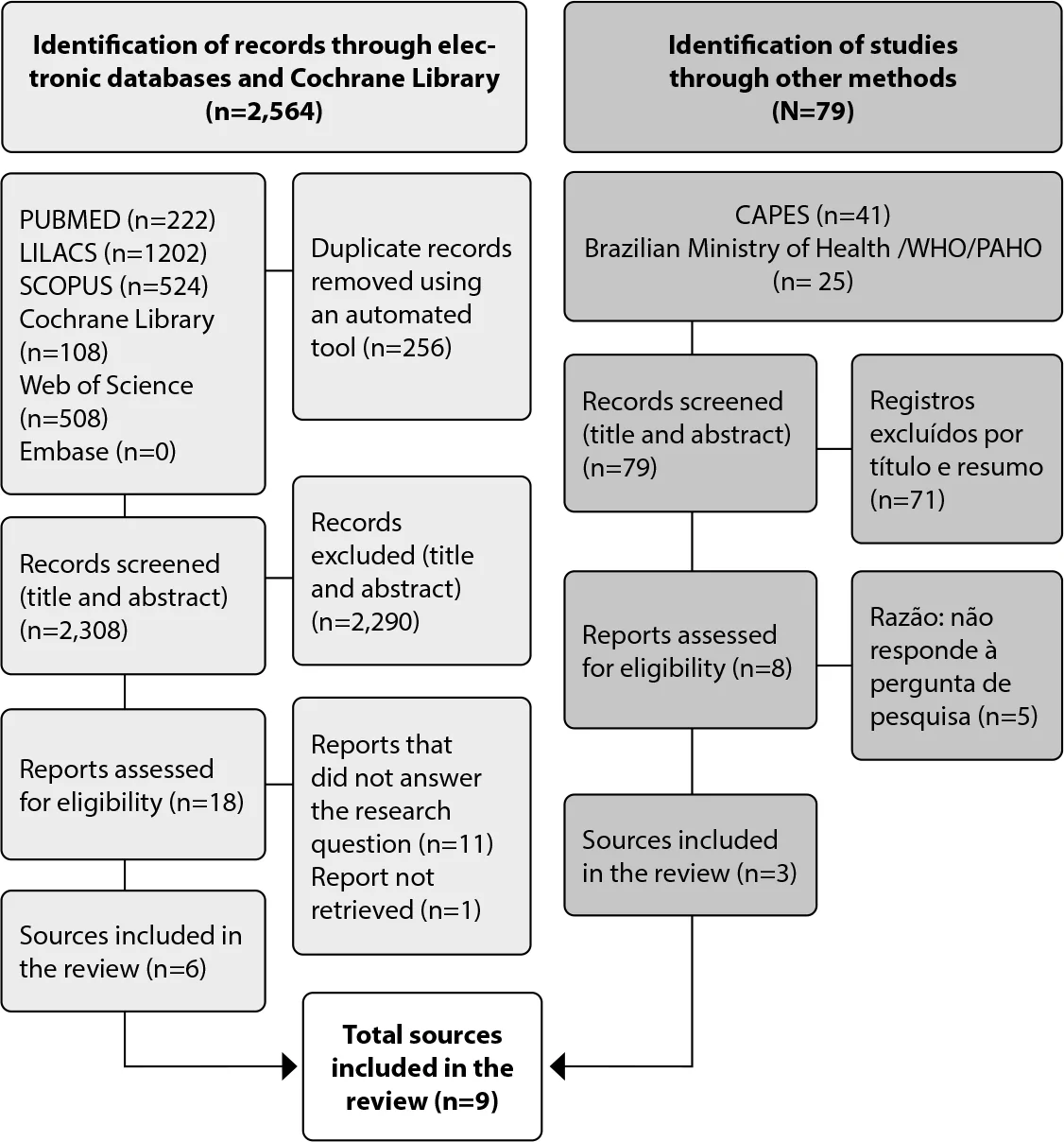
Flow diagram of evidence source selection, Uberaba, Minas Gerais, Brazil, 2025

*Source: Adapted from Page et al., 2021^(14)^.*

A total of 2,643 records were identified: 2,564 records retrieved from the six electronic databases searched and 79 documents identified through other search methods. Of the 2,564 database records, 256 were duplicates and were removed, leaving 2,308 records for title and abstract screening. Of these, 2,290 were excluded for failing to meet the eligibility criteria. Eighteen reports underwent full-text assessment; 12 were excluded based on the predefined criteria, leaving six sources included from database searching. For the other methods search, 79 documents were screened by title and abstract; 71 were excluded, and eight reports were assessed for eligibility. Five were excluded for failing to answer the review question, yielding three additional sources. Thus, the final corpus comprised nine sources included in this scoping review (Figure 1).

All sources included in this review were published in Portuguese (100%): two (22.2%) in 2024, two (22.2%) in 2022, two (22.2%) in 2018, and one (11.1%) each in 2020, 2019, and 2016. In terms of document type, five (55.5%) were journal articles, one (11.1%) was a conference proceedings abstract, one (11.1%) was an e-book, one (11.1%) was a monograph, and one (11.1%) was a law.

In the synthesis, the following study designs were identified: four qualitative studies (44.4%), one descriptive-exploratory study (11.1%), one integrative review (11.1%), and one experience report (11.1%). Because the law and the e-book were not journal articles, they were not classified by study design. When sources were classified by level of evidence according to the JBI methodology^(16)^, three (33.3%) were rated as 3B, three (33.3%) as 5B, two (22.2%) as 4A, and one (11.1%) as 4B. Overall, most sources underscored the importance of integrated work processes.

Chart 2 provides full details for each included source.

**Chart 2:** Summary of included articles and documents, Uberaba, Minas Gerais, Brazil, 2025

Title/year	Objective	Design/Participants (ACE and ACS)	Main findings/Outcomes	Recommendations from the studies	Level of evidence
Ecology of knowledges and territory: integration between ACS and ACE in PHC/ 2024^(17)^	To identify points of convergence in ACSs’ and ACEs’ work processes through the lens of the ecology of knowledges.	Integrative review. Final sample: 18 articles.	This review highlights concrete examples of integrating ACSs’ and ACEs’ activities across diverse settings. It also points to weak links between health surveillance and PHC and describes challenges in these professionals’ day-to-day work processes.	PHC is presented as a central setting for an ecology of practices and knowledge. The studies recommend integrating ACSs and ACEs by enabling mutual observation of each other’s work in shared territories and by developing convergent activities that facilitate knowledge exchange in routine health work.	4A
Integrated work processes between ACS and ACE: an ongoing challenge for health care and health surveillance in Brazil/ 2024^(18)^	To examine how ACSs and ACEs interact at work, identifying challenges and perspectives for integrating teams in disease prevention, health promotion, and health education, based on interviews with course participants.	Qualitative study. Participants: 110 ACSs and ACEs from five regions of Brazil-Northeast (30.8%), South (26%), Southeast (19.2%), North (16.3%), and Central-West (7.7%).	Limited interaction between ACSs and ACEs emerged as a key constraint: collaboration remains sporadic and is typically triggered by one group’s immediate demands. ACSs were reported to interact more frequently with nurses in health units, whereas interaction with physicians was more limited, largely due to high turnover among these professionals. By contrast, the “Saúde com Agente” program was described as a positive driver of integration, increasing access and bringing ACSs and ACEs closer together-especially through in-person activities with course preceptors. Participants noted that this initiative helped build trust and collaboration, enabled experience sharing, and strengthened integration within health services.	The studies also emphasize strengthening integration between ACS and ACE teams to improve health service delivery. The technical courses offered through the “Saúde com Agente” program are described as a powerful tool for advancing this integration. However, additional strategies are needed to expand communication and foster collaboration among these professionals. Establishing a shared physical workspace for both groups may further support network-based coordination and foster more consistent, productive day-to-day interaction.	3B
Territory, neglected diseases, and the work of community and vector control agents/ 2022^(10)^	To characterize ACSs’ and ACEs’ knowledge, practices, and professional experience regarding Hansen’s disease and Chagas disease (DC) during participation in an integrated training workshop within the IntegraDTNs-Bahia project.	Descriptive-exploratory study. Participants: 135 professionals, including 107 ACSs and 28 ACEs.	The study also identified distinct patterns in ACSs’ and ACEs’ knowledge and practice, reflecting fragmented management guidance and work processes among professionals working in the same territory. Differences were observed in access to educational materials and in the implementation of disease-specific prevention and control actions, underscoring the need for clear guidance to support practice integration. Collective narratives highlighted everyday challenges and historical and sociocultural factors, including social stigma. The findings emphasize that health training should go beyond knowledge transmission and foster engagement with local realities. Open dialogue between the two groups (ACS and ACE) was viewed as an opportunity to integrate actions, using the territory as the setting for joint work. This interaction generated new ideas and possibilities, contributing to knowledge generation and improving surveillance and care strategies	One article notes that weak coordination between PHC and endemic disease surveillance and control remains a barrier in endemic territories, underscoring the need for stronger integration between ACEs and ACSs. This collaboration is critical for meeting targets and strengthening cooperative practice. The findings also highlight the need to sustain spaces for joint planning and implementation to make integration routine. Fragmentation of actions and the lack of integrated training further widen this gap, underscoring the urgency of implementing innovative EPS strategies that recognize overlapping risks and vulnerabilities associated with neglected tropical diseases (NTDs).	4ª
Fundamentals of health agents’ work/ 2022^(19)^	To improve performance in both role-specific and shared activities, reinforce cooperative work requirements, and strengthen communication, empathy, and willingness to listen and provide guidance.	E-book.	Integrated and complementary collaboration between ACEs and ACSs within the same territory is essential-and recommended-for appropriately identifying and addressing the community’s health problems, improving the population’s access to health services and public health actions, and preventing disease. Achieving integration requires discussing and planning actions grounded in local realities, strengthening the ability to observe the territory and identify priorities, with a genuine commitment to population health. This accomplishment spans planning and goal setting, defining competencies and responsibilities, and delivering effective care focused on improving quality of life.	Finally, the studies stress the need to integrate PHC and health surveillance through discussion forums, workshops, and joint planning, ensuring that managers and professionals share a clear understanding of the territory they serve. They also emphasize using epidemiologic data to prioritize health problems, move beyond hierarchical arrangements, and adopt genuinely collaborative approaches to controlling health threats. To that end, they recommend including surveillance staff in PHC meetings, involving both sectors in EPS, jointly developing protocols, and entering data into reporting systems. They further highlight the need for coordination between ACSs and ACEs as a strategy to increase the effectiveness of actions in the territory.	5B
Permanent health education as a tool for integration between community health and vector control agents/ 2020^(9)^	To describe the experience of developing permanent health education (EPS) workshops focused on community health promotion with a group of ACSs and ACEs.	Experience report. Two EPS workshops with ACSs and ACEs working in nine basic health units (UBS).	The review identified the impact of EPS in strengthening community health promotion by integrating ACSs and ACEs. Encouraging dialogue between these professionals is a key strategy for bringing the groups closer together and fostering ideas-whether complementary or not-that contribute to the creation of new knowledge.	EPS should be viewed as a strategic tool for promoting integration between ACSs and ACEs, as it strengthens service delivery and keeps the service user at the center, aligning with the need for comprehensive, equitable health promotion actions. Ensuring continuity of EPS spaces, together with ongoing joint planning and implementation of integrated actions between these professionals, is essential to make such practices routine in day-to-day work processes.	5B
Integration between ACS and ACE in health actions in Camaragibe, Pernambuco/ 2019^(20)^	To strengthen integration between ACSs and ACEs by planning health actions for Health Territory 1 in Camaragibe, Pernambuco.	Action research; qualitative study. Participants: 56 professionals.	The impact of EPS on strengthening integrated actions between ACSs and ACEs was evident. Facilitating dialogue between these professionals is essential for bringing the groups closer together, clarifying roles, sharing challenges and solutions, and generating ideas that may or may not be complementary. This process supports the development of new knowledge and practices, strengthening integration.	Investing in EPS is a key strategy for promoting integration between ACSs and ACEs and can serve as a powerful tool for strengthening service delivery. This approach keeps the service user at the center, supporting comprehensive, equitable, and accessible health actions for all.	3B
Experience of integrating ACEs and Family Health Strategy teams in the response to *Aedes aegypti* in Maracanaú, Ceará/ 2018^(21)^	To examine the incorporation of ACEs into ESF teams in Health Surveillance Area II.	Cross-sectional qualitative study. Participants: eight ACEs, five ACSs, six vector control supervisors, and two nurses (n=21).	ACEs’ work in addressing arboviral diseases involves integrated action with ESF teams, enabling early identification of cases and health risks based on information from community members. This integration can have a positive impact on vector control, but sustaining effectiveness requires ongoing monitoring and adaptation as conditions and work processes change.	The study recommends future research focused on this topic to elucidate, through observational studies, the actions involved in integrating ACEs into ESF teams to control arboviral epidemics. It also calls for sustaining this strategy under adequate conditions for a sufficient period to assess which measures are most effective in improving adherence to prevention practices in households and public spaces.	4B
Law No. 13,595, of January 5, 2018/ 2018^(7)^	Amends Law No. 11,350, of October 5, 2006, addressing the revision of roles, working hours and conditions, level of professional training, technical and continuing training courses, and transportation allowance for ACSs and ACEs.	Law.	Not applicable.	ACSs and ACEs will work in an integrated manner, promoting social mobilization through Popular Education in Health (Educação Popular em Saúde) within their geographic areas of practice, especially in the following situations: I - Guiding the community on adopting simple environmental management measures for vector control, individual and collective protection, and other health promotion actions to prevent infectious diseases, zoonoses, vector-borne conditions, and harm caused by venomous animals; II - Planning, scheduling, and carrying out health surveillance activities in coordination with ESF teams; III - Identifying and referring to the designated health unit situations related to environmental factors that affect disease course or are of epidemiologic relevance; IV - Conducting campaigns or joint community task forces to curb transmission of infectious diseases and other health conditions.	5B
Dengue control: consensus developed by vector control agents and community health workers regarding integrated actions/ 2016^(22)^	To analyze the consensus developed by ACSs and ACEs regarding integrated actions following the implementation of the ordinance incorporating ACEs into ESF teams	Analytical qualitative study. Participants: ACSs and ACEs from the 12 neighborhoods in Goiânia’s Northwest Health District.	The study showed that systematically integrating epidemiological and entomological surveillance into PHC strengthens practice and helps avoid duplicated activities. The Collective Subject Discourse (DSC) findings highlighted the roles of ACSs and ACEs and indicated that bringing both groups closer to the ESF team through meetings facilitates knowledge exchange and dengue prevention and control actions. Despite difficulties and questions about effectiveness, ACEs experienced integration more intensely after they began working in PHC. However, the study pointed to shortcomings in communication and in efforts to build awareness and engagement around this process.	The studies also emphasize the importance of investing in training and in opportunities for closer collaboration and familiarization-both among agents and with their supervisors. A structured EPS process that supports and enables the consistent implementation of integrated activities is essential.	3B

## DISCUSSION

This review mapped the published scientific evidence on integration between ACEs and ACSs in the context of PHC. The limited number of articles and documents included indicates a substantial research gap on this topic.

Across the studies reviewed, ACEs were less frequently included as a study population than ACSs. One possible explanation is that ACEs were incorporated into PHC only in 2010 to strengthen health surveillance actions in partnership with ESF teams in Brazil^(6)^.

This underrepresentation of ACEs in the literature also reflects the category’s historical erasure within PHC. While ACSs were progressively incorporated into and regulated within PHC teams-and recognized as a mandatory component of the ESF minimum team since the mid-1990s through Laws No. 10,507/2002 and No. 11,350/2006-ACEs were incorporated later, through Ministerial Ordinance No. 1,007/GM/MS (2010)^(4,6,23)^.

Notably, *Caderno de Atenção Básica* No. 39, which addresses integration between health surveillance and primary care, is grounded in surveillance-related responsibilities for ACEs without including them as members of the PHC team^(24)^. In the 2012 National Primary Care Policy (PNAB), ACEs were not framed in the same way as ACSs and were only mentioned indirectly within endemic disease surveillance and control actions, whereas ACSs were recognized as mandatory members of the ESF minimum team^(25)^. Only in 2017, with the PNAB revision issued through GM/MS Ordinance No. 2,436 (September 21, 2017), did the policy allow for the possibility of incorporating ACEs as an additional member of the primary care team, reinforcing that their inclusion is optional and at the local manager’s discretion rather than part of the core team composition. This decision denotes progress, but also the persistence of a hierarchy between ACSs and ACEs in the PHC context^(3,18)^.

This finding aligns with another review that examined advances in policy guidelines for integrating the work of these professionals. That review found greater publication output on the work of ACSs and ACEs during the 2010s, comprising mainly descriptive and qualitative studies, particularly analyses using the Collective Subject Discourse (DSC) approach. These studies focus primarily on these professionals’ work processes in PHC settings, particularly in the response to arboviral diseases (including dengue) and in *Aedes aegypti* vector control. The review also notes that ACEs’ work tends to prioritize the transmission of technical and scientific information related to controlling infestation sites, vector breeding sites, and zoonotic reservoirs, whereas lay knowledge and popular logics are undervalued^(26)^.

Notably, a specific ordinance intended to regulate integrated actions will require strategies to promote changes in these professionals’ work processes in the setting analyzed. Moving away from entrenched management models and long-standing practices will require ongoing opportunities for reflection involving different stakeholders^(10)^.

Accordingly, existing governance and coordination mechanisms need strengthening to ensure effective integration between ACEs and ACSs and to provide the minimum structural conditions indispensable for adequately performing their roles^(27)^.

The present findings also point to a lack of meaningful, sustained coordination between ACEs and ACSs. The limited number of studies and the lack of broader geographic coverage constrain the generalizability of these findings, making it impossible to conclude that this distance is reproduced nationwide.

Practical integration between ACSs and ACEs remains a challenge for strengthening PHC and for effective health surveillance actions. Recent studies indicate that, although the complementary nature of these two categories is recognized, operational and regulatory gaps persist, hindering coordinated work^(27)^.

Permanent health education strategies, local governance protocols, and shared communication pathways represent concrete opportunities to address current limitations and consolidate more effective collaborative practices^(27,28)^.

An integrative review documented concrete experiences of integrating ACSs’ and ACEs’ actions across different settings, mainly in the response to arboviral diseases^(17)^. Investments in EPS are highlighted as a way to strengthen joint work between ACSs and ACEs^(9)^.

Another study found that when joint activities function as intended, ACSs and ACEs benefit by building dialogue with the population and strengthening ties. According to the authors, both play essential roles in surveillance actions and share responsibility for the health of the populations in their catchment areas. Integrating activities can strengthen work overall, with these professionals complementing each other in territorial practice^(21)^.

One of the included studies identified a long path ahead to achieve more effective epidemic control due to management weaknesses, lack of professional training, and low awareness among the population targeted by public health actions delivered through public services. Ongoing investment in public policies and academic research is therefore needed to reduce the incidence of confirmed cases and prevent deaths-core goals in professional practice^(21)^.

In addition, recent data indicate that, in their day-to-day work during home visits, ACSs and ACEs carry out health promotion and disease prevention actions tailored to local demands and specific needs within their respective territories. According to the agents’ reports, despite the Ministry of Health’s efforts to integrate them into municipal work processes, they still operate independently and engage in collaboration only at specific moments^(29)^.

In the context of the present investigation, the literature also points to substantial challenges to integration, including communication difficulties, lack of joint training, and resistance to change among professionals. The need for ongoing training programs involving both ACSs and ACEs is highlighted as crucial for overcoming barriers and ensuring effective integrated practice^(30)^.

ACEs’ work in addressing arboviral diseases involves integrated actions with ESF teams. This collaboration enables early identification of notified or confirmed cases and related health risks based on information provided by community members; such information is part of ACEs’ characteristic, role-specific responsibilities^(21)^.

In dengue control, integration between ACSs and ACEs is essential, yet it remains marked by major challenges. Key barriers include fragmented regulatory guidance across PHC and health surveillance policies, territory-mapping strategies developed independently by teams, and the absence of effective mechanisms to coordinate field activities. In addition, inadequate infrastructure and limited space for joint planning constrain the implementation of integrated surveillance, vector control, and health education activities^(10,31)^.

At the same time, the setting under analysis also offers meaningful opportunities to strengthen integration between these categories. Recent evidence indicates that a single, shared territory for both groups supports joint action planning, strengthens intersectoral coordination, and increases the effectiveness of local health promotion and disease prevention actions^(31,32)^.

This collaborative work also enables combining ACEs’ technical capacity in vector control with ACSs’ community ties and educational role, resulting in more comprehensive and sustainable strategies for dengue prevention and control^(28)^.

Historically, PHC teams were organized under a predominantly uniprofessional model, which undermines coordination between ACSs and ACEs. From an interprofessional perspective-understood as systematic collaboration across professional categories characterized by communication, shared responsibility, and co-planning-this structural limitation becomes a barrier to the practical integration of these categories within the territory^(33,34)^.

Regarding facilitators and barriers to integration between the two categories, one study found that, on the positive side, reports of teamwork, information sharing, immediate case notification, and prioritization of ESF actions underscore the relevance of integrating ACEs into ESF teams for dengue control. On the negative side, some ACEs and ACSs pointed to ACSs’ workload burden and the lack of intersectoral workflows, effective integration, and autonomy for legal interventions as factors that can undermine ACSs’ credibility with the families for whom they are responsible^(11)^.

In this new sociopolitical context, in which ACEs operate alongside ACSs in a shared territory, it is essential to develop innovative training approaches. Garcia et al.’s proposal^(10)^ guided the review of local strategies, which should be revisited over time and expanded regionally during the initial implementation phase.

That study also showed the need to create forums where health managers can discuss work processes with ACSs and ACEs, clarify areas of shared responsibility, and still respect each profession’s distinct competencies and scope of practice.

A critical step toward overcoming fragmented care is to redesign continuing education processes for health professionals, with a focus on integrating ACSs’ and ACEs’ activities. It is essential to recognize the specificities of each role, while also identifying and valuing the shared and complementary aspects of their practice. Integrated training can foster a broader, more coordinated approach to health care and, in turn, encourage collaboration and teamwork^(21)^.

In this context, investing in EPS with ACSs and ACEs is particularly important. These activities can strengthen problem-solving capacity and alleviate work-related distress. They also foster stakeholder participation and strengthen workforce competencies. Joint work between ACSs and ACEs brings the groups closer together and reinforces that collaboration yields meaningful benefits for the community’s quality of life^(35)^.

Based on the included studies and documents, EPS contributes to greater integration between these two groups of professionals and, consequently, encourages communication and teamwork^(7,9,20,22)^. These EPS activities provide opportunities to refresh, reinforce, and build valuable knowledge in day-to-day work^(27)^. Short-term impacts are already evident, with ACSs and ACEs showing greater motivation to carry out health education activities both in the community and in PHC units, as well as adopting a more discerning approach during home visits.

The law, included as a document, also reinforces the importance of integrating both professional categories within the SUS. By regulating their roles and competencies, it supports professional recognition and acknowledges the essential role these professionals play in health promotion and disease prevention. By establishing guidelines for joint work, the law encourages an integrated approach in which both groups can collaborate in complementary ways to strengthen health surveillance actions, health education, and endemic disease control, contributing to improved living conditions in the communities served^(7)^.

In light of these considerations, integrating ACSs and ACEs within the ESF can strengthen local health actions by enabling a more comprehensive and efficient approach to addressing endemic diseases and other public health issues^(6)^.

### Study limitations

A limitation of this review is that the small number of articles identified may reflect the study’s narrow focus on integration between these two professional categories. Despite this limitation, the chosen scope remains relevant because current guidelines provide for both categories to work jointly.

It is also important to note that ESF is a national initiative that strengthens PHC by organizing care through multiprofessional teams. Internationally, however, ACSs and ACEs are not recognized as distinct categories in the same way as in Brazil. In many countries, similar functions are performed by other health workers without specific regulatory frameworks for these categories. This distinctive feature of the Brazilian system underscores these agents’ national importance: they work directly in health promotion and disease prevention, bringing health services closer to communities and supporting responses to a range of diseases.

In addition, the absence of DeCS and MeSH terms for ACEs limited the search process. Participatory dialogue and measures to strengthen professional recognition may help address this gap and expand the visibility these agents receive for their essential role in Brazilian public health.

### Contributions to the field

This review of integrated work between ACSs and ACEs in the PHC context contributes to the health field by providing an overview of the available scientific evidence. The mapping identified good practices, challenges, and the impacts of cooperation between these two groups of professionals in health promotion, epidemiological surveillance, and endemic disease control. By addressing both the challenges and potential benefits of integration, the study informs managers and policymakers, helping strengthen health work processes and expand access and the quality of care in support of comprehensive care.

## FINAL CONSIDERATIONS

This review identified the available scientific evidence on joint work by ACSs and ACEs in the PHC context, highlighting both barriers to and facilitators of this integration. The findings also highlight a substantial evidence gap in studies examining integration between these professionals in this PHC context.

Based on the included studies, local governance actions are needed to translate the existing regulatory framework into practice and enable effective integration between ACSs and ACEs. Key measures include establishing effective coordination mechanisms, adopting protocols, creating intersectoral communication workflows, defining shared territories for ACSs and ACEs, and implementing interprofessional EPS cycles delivered as a team. The need for adequate infrastructure-especially venues for joint planning-is also emphasized.

Training and capacity-building initiatives that involve both professional categories are recommended, with a focus on communication skills, coordination, and role-specific technical knowledge. In parallel, ongoing monitoring and evaluation systems should be implemented to track the progress and outcomes of integrated actions.

When aligned with PNAB guidelines, these measures can help consolidate a territory-based integrated model and, in turn, strengthen local health surveillance and health promotion. They may also help address longstanding coordination gaps between these categories, underscoring the manager’s central role in organizing this work process. Nevertheless, further studies are needed to examine the intervention strategies used to integrate ACSs’ and ACEs’ work.

Future investigations should also prioritize developing proposals to strengthen this integration, as it represents a promising strategy for reinforcing Brazil’s public health system. Although challenges persist, the available scientific evidence suggests that cooperation between these agents can lead to meaningful improvements in health indicators, provided that appropriate policies and practices are implemented to support integration.

This review may help raise awareness among managers, health professionals, and policymakers about the relevance of this topic, as well as the consequences and impacts of integration for health care delivery in the PHC context, supporting efforts to transform current practice.

## Data Availability

The research data are available within the article.
