# Supplementary material for: Integrated work processes between community health workers and vector control agents: a scoping review
Source: Rev Bras Enferm. 2026 Aug 21;79:e20250170. doi: 10.1590/0034-7167-2025-0170 (PMC13505557; doi:10.1590/0034-7167-2025-0170)
Supplement: Translation

# Translation: Trabalho integrado entre agentes comunitários de saúde e de combate às endemias: revisão de escopo

**DOI:** 10.1590/0034-7167-2025-0170pt

**Authors:** Karina Santos da Silva, Nakita Maria Komori, Fernanda Araújo de Paula Delfino, Kellen Campos Castro Moreira, Bethania Ferreira Goulart

**Affiliations:** I Universidade Federal do Triângulo Mineiro. Uberaba, Minas Gerais, Brasil; II Secrataria Municipal de Saúde. Uberaba, Minas Gerais, Brasil

**Keywords:** Agentes Comunitários de Saúde, Atenção Primária à, Saúde, Trabalho, Pessoal de Saúde, Revisão.

## Abstract

**Objetivos:**

mapear as evidências científicas disponíveis acerca do trabalho integrado entre agentes comunitários de saúde e agentes de combate às endemias, na Atenção Primária à Saúde.

**Métodos:**

revisão de escopo, realizada em novembro de 2024, nas bases de dados *Cochrane Library*, EMBASE, Literatura Latino-Americana e do Caribe em Ciências da Saúde (LILACS)*, National Library of Medicine National Institutes of Health* (PubMed), SCOPUS e *Web of Science*.

**Resultados:**

a amostra obtida, composta por nove documentos, cinco artigos, um resumo em anais de evento, um *e-book*, uma monografia e uma lei, evidenciou a importância da integração profissional para a desconstrução da fragmentação da Atenção Primária à Saúde.

**Considerações Finais:**

a escassez de artigos sugere uma lacuna significativa na temática. A integração de ambas as categorias profissionais pode melhorar as ações de saúde e oferecer uma abordagem mais eficiente no combate às endemias, bem como para outras questões de saúde pública.

## INTRODUÇÃO

A Atenção Primária à Saúde (APS), que organiza e reorienta os serviços de saúde, representa a porta de entrada da população no Sistema Único de Saúde (SUS) e tem como objetivo solucionar os problemas de saúde mais comuns e menos complexos. A Estratégia Saúde da Família (ESF) segue um modelo de assistência baseado em determinantes do processo saúde-doença e considera o indivíduo em seus contextos familiar, social, econômico e cultural, além de incluir ações de vigilância e promoção da saúde. É composta por equipes multiprofissionais da APS, com, no mínimo, um médico, um enfermeiro, um auxiliar ou técnico de enfermagem e agentes comunitários de saúde (ACS), para o território definido^(1,2)^. A ESF necessita de uma composição mínima de quatro ACS para cobertura populacional de 100% da área de abrangência e representam mais de 50% do total de profissionais na ESF^(3)^.

Em 2006, com a promulgação da Lei nº 11.350, as funções dos ACS e dos Agentes de Combate às Endemias (ACE) foram regulamentadas, e já se previa a atuação conjunta de ambos^(4)^.

O papel fundamental dos ACS reside na promoção da saúde, no cotidiano dos usuários e das famílias, em suas áreas de atuação. O trabalho deste profissional, ao estabelecer vínculo com os usuários e suas famílias, facilita as ações e fortalece a mobilização comunitária^(5)^.

Com igual relevância, destaca-se a função dos ACE, os quais trabalham na vigilância, prevenção e controle de doenças relacionadas a fatores de risco ambientais^(6,7)^. Quando atuam de modo integrado ao ACS, contribuem para a implementação de medidas de controle de doenças em surtos e epidemias, como o manejo ambiental e o controle integrado de gestão^(8)^.

O Ministério da Saúde (MS) recomenda a inclusão dos ACE nas equipes da ESF, a fim de obter um trabalho colaborativo dada a necessidade de fortalecer ações de promoção e prevenção à saúde, por meio da compreensão e transformação dos territórios^(6,7)^. Essa nova abordagem para organizar os processos de trabalho nos territórios vinculados aos serviços de saúde exige esforços para superar os modelos desintegrados/fragmentados existentes e possibilita a abertura de novas perspectivas para esses profissionais e gestores de saúde nos diferentes níveis de atenção. Contudo, apesar das diretrizes, em muitas situações, os ACE ainda mantêm suas atividades vinculadas aos Centros de Controle de Endemias ou à estrutura de Vigilância Epidemiológica dos municípios, coordenados por profissionais que não fazem parte diretamente da APS^(9,10)^.

Portanto, a articulação do trabalho entre os ACS e os ACE é essencial para a promoção de um cuidado em saúde ampliado e mais resolutivo. A integração de suas ações permite uma abordagem mais completa e coordenada das necessidades da população, desde a promoção da saúde, prevenção de doenças até a vigilância e controle de endemias. Superar a fragmentação das ações e investir na capacitação integrada dos profissionais é fundamental para fortalecer a atenção à saúde, com vistas à integralidade do cuidado. Tal integração deve preservar as especificidades de cada categoria profissional e permitir o compartilhamento de suas experiências^(10,11)^.

## OBJETIVOS

Mapear as evidências científicas disponíveis acerca do trabalho integrado entre agentes comunitários de saúde e agentes de combate às endemias, na Atenção Primária à Saúde.

## MÉTODOS

### Aspectos éticos

Por se tratar de uma revisão baseada em dados de acesso público, sem a participação direta de seres humanos, a apreciação de Comitê de Ética e Pesquisa foi dispensada.

### Desenho do estudo

Trata-se de uma revisão de escopo, com protocolo de pesquisa aprovado e registrado pelo *Open Science Framework* (DOI 10.17605/OSF.IO/S8ZY2), elaborada conforme as diretrizes do *Joanna Briggs Institute* (JBI)^(12)^ e o quadro teórico proposto por Arksey e O’Malley^(13)^. A revisão cumpriu as recomendações do *checklist* do *Preferred Reporting Items for Systematic Reviews and MetaAnalyses Extension for Scoping Reviews* (PRISMA-ScR)^(14)^, com as seguintes etapas: identificação da questão norteadora; localização de estudos relevantes; seleção de estudos; mapeamento das informações; e agrupamento, resumo e relato dos resultados^(15)^.

### Identificação da questão norteadora

Para nortear a revisão, formulou-se uma questão de pesquisa com base na estrutura mnemônica PCC (População, Conceito e Contexto), sendo P - Agentes Comunitários de Saúde e Agentes de Combate às Endemias, C - Trabalho Integrado e C - Atenção Primária à Saúde. Dessa forma, a pergunta da revisão foi: Quais são as evidências científicas disponíveis acerca do trabalho integrado entre ACS e ACE, na APS?

### Localização de estudos relevantes

A coleta de dados foi realizada em novembro de 2024, em seis bases de dados de referência em saúde: *Cochrane Library*; EMBASE (*Elsevier*); Literatura Latino-Americana e do Caribe em Ciências da Saúde (LILACS); *National Library of Medicine National Institutesof Health* (PubMed), SCOPUS (*Elsevier*) e *Web of Science* (WoS).

A escolha dessas bases fundamentou-se no alto volume de indexação de artigos primários em saúde, no intuito de garantir abrangência e qualidade do estudo. Realizou-se ainda pesquisa de literatura cinzenta nos sites de órgãos oficiais, como a Organização Mundial da Saúde (OMS), a Organização Pan-Americana da Saúde (OPAS) e o Ministério da Saúde (MS), além de busca em catálogos de teses e dissertações, diretrizes e resoluções nacionais e internacionais. Para todas as bases mencionadas, padronizou-se a busca utilizando o Portal de Periódicos da Coordenação de Aperfeiçoamento de Pessoal de Nível Superior (CAPES), por meio da Comunidade Acadêmica Federada (CAFe), com acesso garantido por uma instituição federal de ensino superior.

### Seleção de estudos

A seleção dos estudos para revisão seguiu um processo rigoroso, que incluiu a leitura de títulos, resumos e descritores para identificar aqueles alinhados aos critérios predefinidos.

Para garantir a busca precisa e abrangente de estudos relevantes, foram definidas estratégias específicas para cada fonte de informação. As estratégias de busca combinaram Descritores em Ciências da Saúde (DeCS) e *Medical Subject Headings* (*MeSH Terms)*, com os termos: “Agentes Comunitários de Saúde”, “Atenção Primária à Saúde”, “Trabalho”, “Doenças Endêmicas”, “*Community Health Workers*”, “*Primary Health Care*”, “*Work*” e “*Endemic Diseases*”, com seus respectivos sinônimos, utilizando ainda os operadores *booleanos* “AND” e “OR” (Quadro 1).

**Quadro 1:** Estratégias de busca utilizadas nas bases de dados para revisão de escopo, Uberaba, Minas Gerais, Brasil, 2025

Fonte de informação	Estratégias de buscas avançadas	(n) recuperadas
LILACS	*mh:”*Agentes Comunitários de Saúde*” OR (ACS) OR (Community Health Workers) OR (Agentes Comunitarios de Salud) OR mh:M01.526.485.067.080$ OR mh:N02.360.067.080$ AND mh:”*Atenção Primária à Saúde*” (Primary Health Care) OR (Atención Primaria de Salud) OR mh: N04.590.233.727$ OR mh: D011320$ AND mh:”*Trabalho*” OR (Work) OR (Trabajo) OR mh:I03.946$ OR mh: D014937$ OR mh:”* Doenças Endêmicas*”*	1202
PUBMED	*((“Community Health Worker[Mesh] OR (Community Health Worker) OR (Health Worker, Community) OR (Health Workers, Community) OR (Worker, Community Health) OR (Workers, Community Health) OR (Community Health Aides) OR (Aide, Community Health) OR (Aides, Community Health) OR (Community Health Aide) OR (Health Aide, Community) OR (Health Aides, Community) OR (Barefoot Doctors) OR (Barefoot Doctor) OR (Doctor, Barefoot) OR (Doctors, Barefoot) OR (Family Planning Personnel) OR (Personnel, Family Planning) OR (Planning Personnel, Family) OR (Family Planning Personnel Characteristics) OR (Village Health Workers) OR (Health Workers, Village) OR (Health Worker, Village) OR (Workers, Village Health) OR (Worker, Village Health) OR (Village Health Worker)) AND (“Primary Health Care”[Mesh] OR (Care, Primary Health) OR (Health Care, Primary) OR (Primary Care) OR (Care, Primary) OR (Primary Healthcare) OR (Healthcare, Primary))) AND (“Work”[Mesh] OR (Endemic Diseases) OR (Disease, Endemic) OR (Diseases, Endemic) OR (Endemic Disease))*	222
WEB OF SCIENCE/ SCOPUS	*“Community Health Workers” OR “Community Health Worker” OR “Health Worker, Community” OR “Health Workers, Community” OR “Worker, Community Health” OR “Workers, Community Health” OR “Community Health Aides” OR “Aide, Community Health” OR “Aides, Community Health” OR “Community Health Aide” OR “Health Aide, Community” OR “Health Aides, Community” OR “Barefoot Doctors” OR “Barefoot Doctor” OR “Doctor, Barefoot” OR “Doctors, Barefoot” OR “Family Planning Personnel” OR “Personnel, Family Planning” OR “Planning Personnel, Family” OR “Family Planning Personnel Characteristics” OR “Village Health Workers” OR “Health Workers, Village” OR “Health Worker, Village” OR “Workers, Village Health” OR “Worker, Village Health” OR “Village Health Worker” AND “Primary Health Care” OR “Care, Primary Health” OR “Health Care, Primary” OR “Primary Care” OR “Care, Primary” OR “Primary Healthcare” OR “Healthcare, Primary” AND “Work” OR “Endemic Diseases” OR “Disease, Endemic” OR “Diseases, Endemic” OR “Endemic Disease”*	508/524
EMBASE	*‘Primary health care’/exp AND ‘ Community Health Workers’/exp AND ‘Work’/exp OR ‘ Endemic Diseases’/exp (all feeds)*	0
COCHRANE LIBRARY	*Community Health Workers AND Primary Health Care AND Work OR Endemic*	108
Outros métodos de busca	*“*Agentes Comunitários de Saúde*”, “*Atenção Primária à Saúde*”, “*Trabalho*”, “*Doenças Endêmicas*”, “Community Health Workers”, “Primary Health Care”, “Work” e “Endemic Diseases”*	79
**Data da busca: 16/11/2024.**		

### Mapeamento das informações

Identificou-se um total de 2564 artigos nas seis bases de dados analisadas e 79 documentos nos outros locais de busca. Adotou-se a metodologia PRISMA para sistematizar o processo de inclusão dos estudos^(14)^. Após a etapa de busca, as publicações foram importadas para a plataforma Rayyan, utilizada no processo de triagem na duplicação dos artigos.

Com o intuito de minimizar possíveis vieses de seleção, os estudos foram escolhidos por dois revisores, sendo um principal e outro secundário. Primeiramente, ocorreu a leitura dos títulos e resumos e, de acordo com os critérios de seleção, os revisores selecionaram os estudos de forma independente e, em seguida, compararam os bancos de dados para verificar possíveis divergências. Quando presentes, realizava-se um consenso entre as partes. Um terceiro revisor foi consultado para resolver qualquer discordância.

A extração das informações fundamentou-se no instrumento proposto pelo JBI, que incluiu identificação do artigo, ano e local do estudo, características metodológicas, avaliação do rigor metodológico, apontamentos e discussões sobre o foco temático desta revisão.

### Agrupamento, resumo e relato dos resultados

Para realizar uma análise crítica das fontes, decidiu-se classificar o nível de evidência e o grau de recomendação dos estudos de acordo com a metodologia proposta pelo JBI^(16)^. Os níveis de evidência foram organizados de um a cinco, e os graus de recomendação como A, B ou C. Estudos com maior qualidade metodológica foram classificados como 1A, e os de menor qualidade como 5C. Além disso, verificou-se o fator de impacto dos periódicos nos quais os estudos foram publicados, utilizando informações obtidas na plataforma *Journal Citation Reports* (JCR).

As informações extraídas dos estudos foram organizadas em uma tabela para facilitar a síntese dos dados. Essa organização permitiu uma análise descritiva abrangente, com a apresentação de uma síntese detalhada de cada estudo primário incluído na revisão. Dois pesquisadores experientes trabalharam em conjunto, realizando discussões regulares e aprofundadas para garantir a consistência e a precisão dos resultados.

## RESULTADOS

Foram identificadas 2643 produções, das quais 2564 eram artigos disponíveis nas seis bases eletrônicas analisadas e 79 documentos presentes nos outros locais de busca. Dos 2564 artigos encontrados, 256 eram duplicados e, portanto, considerados apenas uma vez. Na fase de seleção por títulos e resumos, avaliou-se um total de 2308 publicações, com a consequente exclusão de 2290 por não atenderem aos critérios de seleção. Restaram, então, 18 produções para a leitura na íntegra, das quais 12 foram excluídas de acordo com os critérios preestabelecidos, restando seis. Na etapa de identificação de documentos por outros meios, dos 79 resultantes da pesquisa, foram excluídos 71 estudos por títulos e resumos, restando oito para serem avaliados para elegibilidade. Destes, cinco foram excluídos por não responderem à pergunta de pesquisa, incluindo-se, então, três documentos. Dessa forma, o corpus final deste escopo foi composto por nove produções (Figura 1).

**Figura 1:** Fluxograma de seleção de produções científicas, Uberaba, Minas Gerais, Brasil, 2025

*Fonte: Adaptado Page et al., 2021^(14)^.*

Todos os documentos incluídos nesta revisão foram publicados na língua portuguesa (100%), sendo dois (22,2%) no ano de 2024, dois (22,2%) em 2022, dois (22,2%) em 2018 e um (11,1) nos anos de 2020, 2019 e 2016. Ao classificar o tipo de documento encontrado, obteve-se um total de cinco (55,5%) artigos, um resumo em anais de evento (11,1%), um *e-book* (11,1%), uma monografia (11,1%) e uma lei (11,1%).

Ao realizar a síntese dos documentos, constatou-se, quanto ao delineamento do estudo, a presença de quatro estudos qualitativos (44,4%), um estudo descritivo e exploratório (11,1%), uma revisão integrativa (11,1%) e um relato de experiência (11,1%). Destaca-se que, no caso da lei e do *e-book*, por não se configurarem como artigos, não houve classificação do tipo de estudo. Ao delimitar o nível de evidência, conforme a metodologia proposta pelo JBI^(16)^, três (33,3%) possuíam nível 3B, três (33,3%) 5B, dois (22,2%) 4A e um (11,1%) 4B. Verificou-se, portanto, que a maioria das publicações recomenda e confirma a importância da integração dos estudos.

O Quadro 2 apresenta informações completas sobre esses estudos.

**Quadro 2:** Síntese dos artigos e documentos selecionados no estudo, Uberaba, Minas Gerais, Brasil, 2025

Título/ ano	Objetivo	Delineamento/ Participantes (ACE e ACS)	Principais achados/ Desfechos	Recomendações dos estudos	Nível de evidência
Ecologia de saberes e território: integração do ACS e do ACE na Atenção Primária à Saúde/ 2024^(17)^	Compreender as convergências no processo de trabalho dos ACS e ACE à luz da ecologia de saberes.	Revisão integrativa. Obtida amostra final 18 artigos.	A análise dessa revisão destaca experiências concretas de integração entre as atividades dos ACS e ACE em variados contextos. Revela ainda fragilidades na articulação entre a Vigilância em Saúde (VS) e a APS, além de apontar desafios enfrentados no processo de trabalho dos referidos profissionais.	A APS configura-se como espaço central para a ecologia de práticas e saberes. Recomenda-se integrar ACS e ACE mediante a observação do trabalho por ambos, em territórios compartilhados, bem como por meio das atividades convergentes que promovem o intercâmbio de conhecimentos mediados pela saúde.	4A
Trabalho integrado entre Agentes Comunitários de Saúde (ACS) e Agentes de Combates às Endemias (ACE): um desafio a ser superado no que tange às ações de Atenção à Saúde e Vigilância em Saúde no Brasil/ 2024^(18)^	Analisar a interação entre Agentes Comunitários de Saúde (ACS) e Agentes de Combates às Endemias (ACE) no contexto do trabalho, identificando desafios e perspectivas para a integração das equipes na prevenção, promoção e educação da saúde, a partir de entrevistas concedidas por profissionais participantes dos cursos.	Pesquisa qualitativa. Participaram 110 ACS e ACE, das cinco regiões do Brasil: Nordeste (30,8%), Sul (26%), Sudeste (19,2%), Norte (16,3%) e Centro-Oeste (7,7%).	Os resultados indicam um fator limitador na interação entre ACS e ACE, que ocorre de forma esporádica, geralmente motivado por demandas específicas de uma das partes. Verificou-se que os ACS mantêm maior interação com as enfermeiras das Unidades de Saúde, enquanto a interação com os médicos é mais limitada, em razão da alta rotatividade desses profissionais. Por outro lado, destacou-se o impacto positivo do curso “Saúde com Agente”, por ter proporcionado maior acessibilidade e aproximação entre ACS e ACE, especialmente por meio das atividades presenciais realizadas com os preceptores do curso. Essa iniciativa tem fomentado a construção de vínculos de confiança e colaboração, além de favorecer a troca de experiências e integração entre esses atores no âmbito dos serviços de saúde.	Destaca-se a importância de fortalecer a integração entre as equipes de ACS e ACE, com vistas a melhorar o trabalho em saúde. Os cursos técnicos oferecidos pelo Programa Saúde com Agente revelam-se uma potente ferramenta para promover essa integração. Contudo, é essencial investir em outras estratégias que ampliem a comunicação e incentivem o trabalho colaborativo entre esses profissionais. Adicionalmente, a criação de um espaço físico compartilhado por ambas as categorias pode facilitar a articulação em rede, bem como promover uma interação mais consistente e produtiva no cotidiano das práticas de saúde.	3B
Território, doenças negligenciadas e ação de agentes comunitários e de combate a endemias/ 2022^(10)^	Caracterizar conhecimentos, práticas e experiência profissional de agentes comunitários de saúde (ACS) e agentes de controle de endemias (ACE) sobre hanseníase e doença de Chagas (DC), durante participação em oficina de formação integrada no projeto IntegraDTNs-Bahia.	Descritivo e exploratório. Participaram do estudo 135 profissionais, dentre eles 107 ACS e 28 ACE.	O estudo identificou padrões distintos de conhecimento e atuação dos ACS e ACE. Essa diferença reflete diretrizes fragmentadas na gestão e nos processos de trabalho entre profissionais que atuam no mesmo território. Entretanto, constatou-se distinção no acesso a materiais educativos e no desenvolvimento de ações preventivas e de controle para cada doença, evidenciando a necessidade de normativas específicas para a integração das práticas. Os discursos coletivos revelaram desafios cotidianos e aspectos histórico-culturais, incluindo o estigma social. Reforçou-se a importância de uma formação em saúde que transcenda a transmissão de conhecimento e estimule o envolvimento com a realidade local. O diálogo aberto entre os agentes foi reconhecido como uma oportunidade para integrar ações, utilizando o território como espaço de atuação conjunta. Essa interação promoveu novas ideias e possibilidades, contribuindo para a construção de conhecimentos e melhoria das estratégias de vigilância e cuidado.	O artigo sugere que a desarticulação entre a APS e as ações de vigilância e controle de endemias ainda é um obstáculo nos territórios endêmicos, exigindo maior integração entre ACE e ACS. Essa colaboração é essencial para otimizar o alcance das metas e qualificar o trabalho de forma cooperativa. Ademais, destaca-se a importância da continuidade de espaços de planejamento e execução de ações conjuntas, a fim de tornar a integração uma prática rotineira. Evidencia-se ainda que a fragmentação das ações e a falta de capacitação integrada reforçam esse distanciamento, fato que torna premente a implementação de estratégias inovadoras de educação permanente em saúde, que reconheçam a sobreposição de riscos e vulnerabilidades das Doenças Tropicais Negligenciadas (DTN).	4ª
Fundamentos do trabalho do agente de saúde/ 2022^(19)^	Ajudar a aprimorar o desempenho nas atividades específicas e comuns, no sentido de reforçar os requisitos de atuação cooperada e exercitar a capacidade de comunicação, empatia e disponibilidade para a escuta e orientação de ações.	*E-book*	A colaboração integrada e complementar entre os ACE e os ACS, em um mesmo território, é fundamental e recomendável para identificar e abordar adequadamente os problemas de saúde da comunidade; promover o acesso da população a ações e serviços de saúde e prevenir doenças. A integração exige o debate de ações com base na realidade local, de modo a desenvolver a capacidade de observar o território e reconhecer prioridades, comprometendo-se, verdadeiramente, com a saúde da população. Isso envolve desde o planejamento e definição de metas, competências e responsabilidades, até a oferta de cuidado efetivo às pessoas, com foco na melhoria da qualidade de vida.	Destaca-se a importância de integrar a Atenção Básica e a Vigilância em Saúde por meio de discussão, escritórios e planejamento conjunto, garantindo que gestores e profissionais compreendam o território de atuação. Salienta-se ainda a necessidade do uso da epidemiologia na priorização de problemas de saúde e no sentido de superar a lógica hierárquica e promover uma abordagem de fato colaborativa no controle de agravos. Para tanto, recomenda-se a inclusão de trabalhadores da vigilância nas reuniões da Atenção Básica, bem como a participação de ambos os setores na educação permanente, no desenvolvimento de protocolos e na alimentação de sistemas de notificação. Adicionalmente, enfatiza-se a articulação entre ACS e ACE como estratégia para aumentar a efetividade das ações no território.	5B
Educação permanente como ferramenta de integração entre agentes de saúde e de endemias 2020^(9)^	Relatar a experiência de desenvolvimento de oficinas de educação permanente em saúde (EPS) com foco na promoção da saúde para a comunidade com um grupo formado por agentes comunitários de saúde (ACS) e agentes de combate às endemias (ACE).	Relato de experiência. Duas oficinas com os ACS e ACE cadastrados em nove UBS.	Identificou-se o impacto da EPS no fortalecimento das ações de promoção da saúde na comunidade por meio da integração entre ACS e ACE. O incentivo ao diálogo entre esses profissionais é uma estratégia fundamental para aproximar as categorias e favorecer o surgimento de ideias complementares ou não, mas que contribuam para a construção de novos saberes.	A EPS deve ser vista como uma ferramenta estratégica para promover a integração entre ACS e ACE, pois qualifica o serviço e tem como foco principal o usuário, que demanda ações de promoção da saúde de maneira integral e equitativa. É fundamental assegurar a continuidade dos espaços de EPS, bem como do planejamento e da execução de ações integradas entre esses profissionais, para que tais práticas se consolidem como parte da rotina no processo de trabalho.	5B
Integração entre Agentes Comunitários de Saúde (ACS) e de Combate às Endemias (ACE) nas ações de saúde do município de Camaragibe - PE/ 2019^(20)^	Fortalecer a integração entre Agentes Comunitários de Saúde (ACS) e de Combate às Endemias (ACE) por meio do planejamento para realização das ações de saúde do território de saúde 1 no município de Camaragibe - PE.	Método de pesquisa-ação, pesquisa qualitativa. Participaram do estudo 56 profissionais.	O impacto da EPS no fortalecimento das ações integradas entre ACS e ACE foi evidente. Promover o diálogo entre esses profissionais configura uma estratégia essencial para aproximar as categorias, permitir a descoberta de funções, o compartilhamento de desafios e soluções, além do desenvolvimento de ideias que podem ser complementares ou não. Esse processo favorece a construção de novos conhecimentos e práticas, fortalecendo a integração.	Investir na EPS representa uma estratégia fundamental para promover a integração entre ACS e ACE, configurando-se como uma ferramenta potencial para a qualificação do serviço. Essa iniciativa posiciona o usuário como a principal referência, garantindo ações de saúde integrais, equitativas e acessíveis a todos.	3B
Experiência da integração entre agentes de endemias e equipes de saúde da família no enfrentamento ao *Aedes aegypti* no município de Maracanaú-CE/ 2018^(21)^	Analisar a inserção dos ACE nas ESF na Área de Vigilância à Saúde II.	Estudo transversal e qualitativo. Oito ACE, cinco ACS, seis supervisores de endemias e dois enfermeiros, totalizando 21 participantes.	O trabalho do ACE no combate às arboviroses envolve ações integradas com as equipes da ESF, o que permite a identificação precoce de casos e riscos à saúde, com base em informações da população. Essa integração exerce impacto positivo no controle do vetor, mas requer acompanhamento contínuo das mudanças e dos processos envolvidos para garantir sua efetividade.	A pesquisa propõe a realização de estudos futuros focados nesta temática, com o objetivo de elucidar, por meio de pesquisas observacionais, as ações que envolvem a integração dos ACE, nas ESF, para o controle de epidemias de arboviroses. Além disso, busca-se dar continuidade a essa estratégia, garantindo as condições necessárias por tempo suficiente para avaliar quais medidas são mais eficazes para adesão às práticas de prevenção em domicílios e locais públicos.	4B
Lei nº 13.595, de 5 de janeiro de 2018/ 2018^(7)^	Altera a Lei nº 11.350, de 5 de outubro de 2006, para dispor sobre a reformulação das atribuições, a jornada e as condições de trabalho, o grau de formação profissional, os cursos de formação técnica e continuada e a indenização de transporte dos Agentes Comunitários de Saúde e Agentes de Combate às Endemias.	Lei	Não se aplica.	Os ACS e os ACE atuarão de maneira integrada, promovendo mobilizações sociais, por meio da Educação Popular em Saúde, dentro de suas áreas de atuação geográfica, especialmente nas seguintes situações: I - Orientar a comunidade sobre a adoção de medidas simples de manejo ambiental para controle de vetores, proteção individual e coletiva, além de outras ações de promoção da saúde, a fim de prevenir doenças infecciosas, zoonoses, agravos por transmissão vetorial e danos causados por animais peçonhentos; II - Planejar, programar e executar atividades de vigilância em saúde, em articulação com as equipes de saúde da família; III - Identificar e encaminhar para a unidade de saúde de referência situações relacionadas a fatores ambientais que interfiram no curso de doenças ou possuam relevância epidemiológica; V - Realizar campanhas ou mutirões para combate à transmissão de doenças infecciosas e outros agravos.	5B
Controle da dengue: os consensos produzidos por Agentes de Combate às Endemias e Agentes Comunitários de Saúde sobre as ações integradas/ 2016^(22)^	Analisar os consensos produzidos pelos ACS e ACE sobre essas ações integradas com a implantação da Portaria de Incorporação dos ACE nas equipes da Estratégia Saúde da Família (ESF).	Estudo analítico, qualitativo. Participaram ACS e ACE dos 12 bairros pertencentes ao Distrito Sanitário Noroeste de Goiânia.	O estudo mostrou que a integração sistemática da vigilância epidemiológica e entomológica na Atenção Básica fortalece o trabalho, evitando a duplicidade de ações. Os Discursos do Sujeito Coletivo (DSC) destacaram o papel dos ACS e ACE e evidenciaram que a aproximação entre ambos com a equipe da ESF, por meio de reuniões, favorece a troca de conhecimentos e as ações de prevenção e controle da dengue. Apesar das dificuldades e questionamentos sobre sua eficácia, a integração foi mais intensamente vivenciada pelo ACE, que passou a atuar na APS. No entanto, o estudo apontou falhas na comunicação e sensibilização desse processo.	Destaca-se a importância de investir em capacitação e em momentos de maior proximidade e familiarização, tanto entre os agentes quanto com seus supervisores. Torna-se indispensável um processo de EPS que promova e facilite a execução de atividades integradas de forma consistente.	3B

## DISCUSSÃO

Esta revisão mapeou as evidências científicas já publicadas sobre a integração entre os ACE e os ACS no contexto da APS. A baixa quantidade de artigos e documentos selecionados nesta revisão evidencia uma importante lacuna no que se refere à investigação da temática.

Quando avaliado o quantitativo de ACE, como população da pesquisa, diante da quantidade de ACS, constatou-se ser inferior nos estudos analisados. Isso pode ser explicado pelo fato de que esses profissionais foram incorporados na APS apenas em 2010, na época com o propósito de fortalecer as ações da VS em parceria com as equipes da ESF^(6)^ no cenário brasileiro.

Esse déficit de ACE verificado nas pesquisas reflete ainda um apagamento histórico da categoria dos ACE no âmbito da APS. Enquanto os ACS foram progressivamente alocados e normatizados nas equipes da APS, bem como reconhecidos como componente obrigatório da equipe da ESF desde meados da década de 1990, por meio das Leis nº 10.507/2002 e nº 11.350/2006, os ACE foram incorporados apenas mais tardiamente, através da Portaria Ministerial 1.007/GM/MS 2010^(4,6,23)^.

Salienta-se que o Caderno de Atenção Básica nº 39, voltado à integração entre VS e Atenção Básica, fundamenta-se em atribuições de vigilância para o ACE, sem incluí-lo como membro da equipe da APS^(24)^. Na Política Nacional de Atenção Básica (PNAB), publicada em 2012, os ACE não receberam o mesmo enquadramento que os ACS, e foram apenas mencionados de forma indireta nas ações de vigilância e controle de endemias. Já os ACS foram reconhecidos como membros obrigatórios da equipe mínima da ESF^(25)^. Apenas em 2017, na revisão da PNAB, promulgada por meio da Portaria GM/MS nº 2.436 de 21/09/2017, que se evidenciou a possibilidade de incorporação do ACE como integrante na equipe de Atenção Básica, de forma adicional, reforçando seu caráter opcional conforme definição do gestor local e não na composição básica, o que denota um avanço, mas, também, a persistência de hierarquia entre ACS e ACE, no cenário da APS^(3,18)^.

O referido resultado tem convergência com outra revisão, cujo intuito foi investigar os avanços das diretrizes políticas para a integração do trabalho dos profissionais supracitados. Constatou-se maior produção referente à atuação dos ACS e ACE, na década de 2010, com estudos descritivos e qualitativos, especialmente análises do Discurso do Sujeito Coletivo (DSC). Tais estudos discutem, sobretudo, o processo de trabalho dos referidos profissionais na APS, no enfrentamento às arboviroses, à dengue e ao controle vetorial do *Aedes aegypti*. Há também pesquisas sobre a atuação dos ACE no controle da doença de Chagas, malária e zoonoses. Discute-se ainda que, na atuação dos ACE, predomina a transmissão de informações técnico-científicas relacionadas ao controle de focos, criadouros de vetores e reservatórios de zoonoses, enquanto os saberes e a lógica popular são pouco valorizados^(26)^.

Destaca-se que a normatização em portaria específica para o desenvolvimento de ações integradas necessita de estratégias para promover mudanças no processo de trabalho dos profissionais no cenário analisado. Logo, a ruptura com modelos de gestão e práticas enraizadas requer espaços contínuos de reflexão, envolvendo os diversos atores^(10)^.

Dessa forma, evidencia-se a necessidade de aprimorar os mecanismos de governança e de coordenação existentes, de modo a assegurar uma efetiva integração entre os agentes e garantir condições estruturais mínimas indispensáveis ao adequado desempenho de suas atribuições^(27)^.

Os resultados da presente investigação mostram ainda falta de aproximação efetiva entre as categorias de ACE e ACS na atualidade. O número restrito de estudos e a ausência de abrangência geográfica mais ampla limitam a possibilidade de generalização desses achados, não sendo possível afirmar, de forma conclusiva, que tal distanciamento se reproduza em todo o território nacional.

A integração prática entre os ACS e os ACE permanece um desafio para o fortalecimento da APS e para a efetividade das ações de VS. De acordo com estudos recentes, embora haja reconhecimento da complementaridade entre as referidas categorias, persistem fragilidades operacionais e normativas que dificultam o trabalho articulado^(27)^.

Estratégias de educação permanente, protocolos de governança local e fluxos de comunicação compartilhados representam oportunidades concretas para superar as limitações atuais e consolidar práticas colaborativas mais eficazes^(27,28)^.

Estudo de revisão integrativa evidenciou experiências concretas de integração das ações de ACS e ACE em diferentes cenários, principalmente no enfrentamento das arboviroses^(17)^. Investimentos em EPS são destacados como forma de potencializar o trabalho conjunto entre ACS e ACE^(9)^.

Em outro estudo, verificou-se que quando as atividades conjuntas funcionam, ACS e ACE são beneficiados em termos de construção de diálogo com a população e criação de vínculo. Segundo os autores, ambos desempenham papéis fundamentais no desenvolvimento das ações de vigilância e são corresponsáveis pela saúde da população de suas áreas de abrangência. A integração das atividades deve potencializar o trabalho, e os profissionais se complementam na atuação no território^(21)^.

Identificou-se, em um dos estudos selecionados, a existência de uma longa trajetória a ser percorrida para alcançar um controle mais eficaz das epidemias, devido às fragilidades na gestão, à falta de capacitação dos profissionais e à baixa conscientização da população beneficiária das ações de saúde ofertadas pelo serviço público. Há, portanto, necessidade contínua de investimento em políticas públicas e pesquisas acadêmicas, com vistas a reduzir a incidência de casos confirmados e prevenir óbitos, que são objetivos primordiais na prática profissional^(21)^.

Ademais, dados recentes indicam que, no cotidiano de trabalho dos ACS e ACE, durante as visitas domiciliares, eles executam ações de promoção e prevenção de doenças adaptadas conforme a demanda e as necessidades específicas, nos respectivos territórios de atuação. De acordo com os relatos dos agentes, apesar de todos os esforços do MS para integrá-los às práticas dos municípios visitados, eles ainda operam de forma independente e participam de colaborações conjuntas apenas em momentos específicos^(29)^.

No contexto da presente investigação, a literatura também revela desafios significativos na integração, inclusive dificuldades de comunicação, falta de treinamento conjunto e resistência à mudança por parte dos profissionais. A necessidade de programas de capacitação contínua, que envolvam tanto ACS quanto ACE, é destacada como crucial para superar barreiras e garantir uma atuação integrada eficaz^(30)^.

O trabalho do ACE, com relação ao combate às arboviroses, envolve um conjunto de ações integradas com as equipes da ESF. Essa colaboração possibilita a identificação precoce de informações sobre casos notificados ou confirmados de pessoas em situação de risco iminente à saúde, as quais são obtidas da população e constituem atribuições características e específicas do ACE^(21)^.

No âmbito do enfrentamento da dengue, a integração entre o ACS e o ACE mostra-se essencial, porém ainda marcada por importantes desafios. Entre os principais entraves, há a fragmentação normativa entre as políticas de APS e VS, as estratégias de mapeamento territorial adotadas pelas equipes que se desenvolvem de forma independente e a ausência de mecanismos efetivos de coordenação das ações de campo. Adicionalmente, a insuficiência de infraestrutura e de espaços de planejamento conjunto limita a realização de atividades integradas de vigilância, controle vetorial e educação em saúde^(10,31)^.

Por outro lado, o cenário em foco também apresenta oportunidades significativas para o aprimoramento da integração entre essas categorias. Evidências recentes indicam que um território único e compartilhado entre ambas contribui para o planejamento conjunto das ações, o fortalecimento da articulação intersetorial e a efetividade das ações de promoção e prevenção em saúde no âmbito local^(31,32)^.

Este trabalho colaborativo permite ainda combinar a capacidade técnica dos ACE no controle vetorial com o vínculo comunitário e o papel educativo dos ACS, resultando em estratégias mais abrangentes e sustentáveis de prevenção e combate à dengue^(28)^.

Historicamente, as equipes da APS foram organizadas sob um modelo predominantemente uniprofissional, o que desfavorece a articulação entre ACS e ACE. Considerando-se a interprofissionalidade, entendida como a colaboração sistemática entre diferentes categorias profissionais com comunicação, corresponsabilidade e coplanejamento, a limitação estrutural revela-se um entrave à integração prática dessas categorias no território^(33,34)^.

Em relação aos fatores facilitadores e dificultadores para a integração entre as duas categorias, pesquisa mostrou que, quanto aos aspectos positivos, os relatos sobre trabalho em equipe, compartilhamento de informações, notificação imediata de casos e priorização das ações da ESF indicam a relevância da integração dos ACE junto às equipes da ESF para as ações de controle da dengue. No que se refere aos aspectos negativos, sob a perspectiva de alguns ACE e ACS, o acúmulo de funções dos ACS e a ausência de fluxo intersetorial, de integração efetiva e de autonomia para intervenções legais podem comprometer a credibilidade do trabalho realizado pelos agentes comunitários junto às famílias sob suas responsabilidades^(11)^.

Diante deste novo contexto sociopolítico, no qual os ACE operam em um território compartilhado com os ACS, torna-se fundamental desenvolver processos de formação inovadores. A proposta delineada no estudo de Garcia *et al*.^(10)^ serviu como guia e fonte de inspiração para a revisão de estratégias locais, as quais devem ser revisitadas em diferentes momentos e expandidas regionalmente, em um primeiro estágio de implementação.

A pesquisa supracitada evidenciou ser essencial criar espaços e oportunidades para que os gestores de saúde discutam práticas com os ACS e os ACE, a fim de identificar responsabilidades que possam ser compartilhadas, enquanto respeitam as habilidades e as particularidades características de cada uma das profissões.

Um dos aspectos críticos para superar essa fragmentação da assistência é a reformulação dos processos de formação continuada dos profissionais de saúde, com foco na integração das atividades dos ACS e ACE. Necessário reconhecer as especificidades de cada função, mas também identificar e valorizar os aspectos comuns e complementares de suas práticas. Capacitações integradas podem promover uma visão mais ampla e coordenada do cuidado em saúde e, assim, incentivar a colaboração e o trabalho em equipe^(21)^.

Nesse contexto, destaca-se a importância do investimento em EPS com os ACS e os ACE. Tais atividades têm o potencial de aumentar a capacidade de resolução dos problemas e de aliviar os sentimentos de sofrimento relacionados ao trabalho. Elas se consolidam como uma estratégia promotora da participação dos diversos atores envolvidos e de suas qualificações. O trabalho conjunto entre ACS e ACE favorece a aproximação e a compreensão de que a colaboração proporciona benefícios significativos para a qualidade de vida da comunidade^(35)^.

De acordo com a análise dos estudos/documentos encontrados, a realização de EPS proporciona maior integração entre esses profissionais e, por conseguinte, estimula a comunicação e o trabalho em equipe^(7,9,20,22)^. As EPS oferecidas funcionam como ferramentas de atualização, reforço e aprendizado de novos conhecimentos úteis no cotidiano de tais profissionais^(27)^. Os impactos a curto prazo dessa educação continuada já são perceptíveis, com ACS e ACE demonstrando maior motivação para realizar ações de educação em saúde, tanto na comunidade quanto na unidade de saúde da APS, além de adotarem um olhar mais criterioso durante as visitas domiciliares.

Destaca-se ainda que a lei encontrada como documento reforça a importância da integração entre ambas as categorias profissionais, no âmbito do SUS. Essa legislação promove a valorização desses profissionais ao regulamentar suas atribuições e competências, reconhecendo o papel essencial por eles desempenhado na promoção da saúde e na prevenção de doenças. Ao estabelecer diretrizes para a atuação conjunta, a lei incentiva uma abordagem integrada, na qual ambos podem colaborar de forma sinérgica para fortalecer ações de VS, educação sanitária e controle de doenças endêmicas, contribuindo para a melhoria das condições de vida das comunidades atendidas^(7)^.

Diante do exposto, a integração dos ACS e ACE, na ESF, pode potencializar as ações de saúde, por proporcionar uma abordagem mais abrangente e eficiente na luta contra as endemias e outras questões de saúde pública^(6)^.

### Limitações do estudo

Como limitações do estudo, destaca-se que o número reduzido de artigos encontrados pode decorrer da natureza do foco da investigação, fundamentado na integração das duas categorias profissionais apenas. Em que isso pese como limitação, acredita-se na relevância do recorte realizado, pois está previsto nas diretrizes que ambas atuem de forma conjunta.

Ressalta-se que a ESF é uma iniciativa nacional que fortalece a APS, ao estruturar o cuidado por meio de equipes multiprofissionais. No entanto, internacionalmente, as categorias de ACS e ACE não são exclusivamente reconhecidas como ocorre no Brasil. Em muitos países, funções semelhantes são desempenhadas por outros profissionais de saúde, sem uma regulamentação específica para essas categorias. Essa particularidade do sistema brasileiro destaca a importância desses agentes no contexto nacional, onde atuam diretamente na promoção da saúde e prevenção de doenças, aproximando os serviços de saúde da população e contribuindo para o enfrentamento de diversas doenças.

Além disso, sublinha-se a ausência dos termos “Desc” e “*Mesh*” para ACE como uma limitação para a pesquisa. Por meio de um diálogo participativo e da implementação de medidas de valorização profissional, é possível superar essa lacuna e garantir que esses agentes recebam o reconhecimento e a visibilidade que merecem pelo papel fundamental por eles desempenhado na saúde pública brasileira.

### Contribuições para a área

A realização de uma revisão sobre o trabalho integrado entre ACS e ACE, no contexto da APS, contribui significativamente para a área da saúde, pois fornece um panorama das evidências científicas disponíveis sobre essa temática. Esse mapeamento permitiu identificar boas práticas, desafios e impactos de cooperação entre esses profissionais na promoção da saúde, vigilância epidemiológica e controle de doenças endêmicas. Ao abordar os desafios e potenciais benefícios da referida integração, o estudo oferece subsídios para gestores e formuladores de políticas públicas, no sentido de colaborar para a qualificação do trabalho em saúde e ampliação do acesso e qualidade, com vistas à integralidade da assistência.

## CONSIDERAÇÕES FINAIS

A presente revisão possibilitou identificar as evidências científicas disponíveis acerca do trabalho conjunto realizado por ACS e ACE, no contexto da APS, destacando aspectos dificultadores e facilitadores para esta integração. Os resultados revelam uma lacuna significativa na literatura referente a estudos que investiguem a integração dos referidos profissionais, no contexto em foco.

De acordo com os resultados dos estudos analisados, as ações de gestão local são reconhecidas como necessárias para concretizar as normativas específicas que possibilitem a integração efetiva entre os ACS e os ACE. Medidas como a incorporação de mecanismos efetivos de coordenação, instituição de protocolos, fluxos de comunicação intersetorial, delimitação dos territórios de atuação comum entre as categorias dos ACS e dos ACE e implementação de ciclos de EPS realizadas em equipe interprofissional são necessárias para incentivar a integração das referidas categorias. Destaca-se ainda a necessidade de infraestrutura para ofertar espaços de planejamento conjunto.

Recomenda-se promover treinamentos e capacitações que envolvam ambas as categorias profissionais, focando em habilidades de comunicação, coordenação e conhecimentos técnicos específicos. Paralelamente, sugere-se implementar sistemas de monitoramento e avaliação contínuos para acompanhar o progresso e os resultados das ações integradas.

Tais medidas, quando articuladas às diretrizes da PNAB, podem favorecer a consolidação de um modelo territorial integrado e, portanto, fortalecer a vigilância e a promoção da saúde em nível local, bem como contribuir para superar as fragilidades históricas de articulação entre as categorias, o que destaca o importante papel do gestor na organização desse processo de trabalho. Entretanto, mais estudos são necessários para explorar as estratégias de intervenção utilizadas para a integração do trabalho entre o ACS e o ACE.

Recomenda-se ainda a realização de investigações futuras que invistam em desenvolver propostas para aprimoramento dessa integração, por representar uma estratégia promissora para fortalecer o sistema de saúde pública no Brasil. Embora existam desafios, as evidências científicas sugerem que a cooperação entre os agentes supracitados pode levar a melhorias significativas nos indicadores de saúde, desde que sejam implementadas políticas e práticas adequadas para apoiar essa integração.

Esta revisão poderá contribuir para sensibilizar a gestão, os profissionais de saúde e os formuladores de políticas sobre a relevância do tema, as consequências e os impacto na assistência à saúde, no contexto da APS, com vistas à transformação da realidade.

## Data Availability

Os dados de pesquisa estão disponíveis no corpo do artigo.
